# Malnutrition: Percentage and Association with Prognosis in Patients Hospitalized for Coronavirus Disease 2019

**DOI:** 10.3390/nu12123679

**Published:** 2020-11-28

**Authors:** Lucie Allard, Elise Ouedraogo, Julie Molleville, Helene Bihan, Bénédicte Giroux-Leprieur, Angela Sutton, Camille Baudry, Constant Josse, Morgane Didier, David Deutsch, Olivier Bouchaud, Emmanuel Cosson

**Affiliations:** 1 Department of Endocrinology-Diabetology-Nutrition, AP-HP Avicenne Hospital, 93000 Bobigny, France; lucie.allard@aphp.fr (L.A.); julie.molleville@aphp.fr (J.M.); helene.bihan@aphp.fr (H.B.); cabaudry@ghpsj.fr (C.B.); 2 Department of Infectious Disease, AP-HP Avicenne Hospital, 93000 Bobigny, France; elise.ouedraogo@aphp.fr (E.O.); olivier.bouchaud@aphp.fr (O.B.); 3 Department of Rehabilitation Nutrition-Obesity, AP-HP René Muret Hospital, 93270 Sevran, France; 4 LEPS (Laboratoire Educations et Pratiques de Santé) EA 3412, Université Paris 13, 93000 Bobigny, France; 5 Department of Internal Medicine, AP-HP Avicenne Hospital, 93000 Bobigny, France; benedicte.giroux-leprieur@aphp.fr; 6 Laboratory for Vascular Translational Science (LVTS), Inserm U1148 Groupe Biothérapies et Glycoconjugués/Université Paris 13—Sorbonne Paris Nord, 93000 Bobigny, France; angela.sutton@aphp.fr; 7 Department of Biology Laboratory, AP-HP Avicenne Hospital, 93000 Bobigny, France; 8 eXYSTAT, 92240 Malakoff, France; constant.josse@exystat.com; 9 Department of Respiratory Disease, AP-HP Avicenne Hospital, 93000 Bobigny, France; morgane.didier@aphp.fr; 10 Department of Gastroenterology and Digestive Oncology, AP-HP Avicenne Hospital, 93000 Bobigny, France; david.deutsch@aphp.fr; 11 Equipe de Recherche en Epidémiologie Nutritionnelle (EREN), UMR U1153 Inserm/U1125 Inrae/Cnam/Université Paris 13—Sorbonne Paris Nord, Centre de Recherche en Epidémiologie et Statistiques—Université de Paris (CRESS), EREN, SMBH PARIS 13, 93000 Bobigny, France

**Keywords:** malnutrition, nutritional risk, COVID-19, prognosis

## Abstract

Previous studies have found a correlation between malnutrition and prognosis in respiratory infections. Our objectives were to determine (i) the percentage of malnutrition, and (ii) its prognosis in patients admitted for coronavirus disease 2019 (COVID-19). In this monocentric retrospective study, we consecutively included all adult patients presenting with acute COVID-19 between 9 April and 29 May 2020. Malnutrition was diagnosed on low body mass index (BMI) and weight loss ≥ 5% in the previous month and/or ≥ 10% in the previous six months. The Nutritional Risk Index (NRI) defined nutritional risk. Severe COVID-19 was defined as a need for nasal oxygen ≥ 6 L/min. We enrolled 108 patients (64 men, 62 ± 16 years, BMI 28.8 ± 6.2 kg/m^2^), including 34 (31.5%) with severe COVID-19. Malnutrition was found in 42 (38.9%) patients, and moderate or severe nutritional risk in 83 (84.7%) patients. Malnutrition was not associated with COVID-19 severity. Nutritional risk was associated with severe COVID-19 (*p* < 0.01; *p* < 0.01 after adjustment for C reactive protein), as were lower plasma proteins, albumin, prealbumin, and zinc levels (*p* < 0.01). The main cause of malnutrition was inflammation. The high percentage of malnutrition and the association between nutritional risk and COVID-19 prognosis supports international guidelines advising regular screening and nutritional support when necessary.

## 1. Introduction

In clinical practice, coronavirus disease 2019 (COVID-19) could be associated with a high prevalence of malnutrition as a consequence of severe acute inflammatory status and decreased appetite, resulting in a negative nutritional balance [1]. Even though a high prevalence of malnutrition has been reported in elderly or critically ill patients [2], we have not found any study evaluating malnutrition in every hospitalized patient out of the intensive care unit (ICU).

Several risk factors for severe COVID-19 pneumonia have been identified, including older age, male gender, and metabolic comorbidities [3,4,5,6,7,8]. Malnutrition might also be a prognostic factor for COVID-19–related increased morbidity and mortality [9], and guidelines have been issued about the need for nutritional support [1,10,11,12,13,14]. For example, it was observed that low plasma prealbumin level predicts progression to acute respiratory distress syndrome [5], but an association between malnutrition and the severity of COVID-19 has never been demonstrated.

Our objectives were to establish (i) the percentage of malnutrition in patients admitted in COVID-19 units (excluding intensive care units (ICU)) and (ii) the prognostic value of malnutrition parameters. Our hypothesis was that malnutrition is associated with poor prognosis in patients hospitalized for acute COVID-19. Indeed, malnutrition has been associated with worse prognosis of respiratory bacterial infections [15,16], influenza [17], or tuberculosis [18] by altering the immune system [19] and the muscular capacity through sarcopenia.

## 2. Materials and Methods

### 2.1. Study Design and Setting

We conducted a retrospective observational study in Avicenne Hospital, Bobigny, France. We included patients admitted between 9 April 2020 and 29 May 2020. This was just after the recommendations of the French-speaking Society for Clinical Nutrition and Metabolism (SFNCM) for the screening and treatment of malnutrition in patients hospitalized for COVID-19 [20] were implemented in our institution. In our university hospital and the Assistance Publique—Hôpitaux de Paris hospitals in general, patients at admission are informed that their medical records could be used for research, unless they oppose. A local ethic committee gave an agreement for file analysis (CLEA-2020-121). We analyzed the data anonymously. No patient opposed.

### 2.2. Participants

All consecutive adult patients were included after being admitted for COVID-19 in one of the four dedicated departments, either directly or through the emergency department. Therefore, we included patients with infection requiring hospitalization but not in an ICU. COVID-19 was confirmed by nasal polymerase chain reaction (PCR) and/or COVID-19 pneumonia criteria on chest computed tomography (CT). Non-inclusion criteria were (i) enteral nutrition at baseline, (ii) major preexisting disease with hypoprotidemia (uncontrolled cirrhosis, nephrotic syndrome), (iii) pregnancy, and (iv) no expected benefit of a transfer into the ICU in case of disease worsening.

### 2.3. Variables: Malnutrition

As defined by the French Health Authority [21], malnutrition was diagnosed if at least one of the three following criteria was present: body mass index (BMI) < 18.5 kg/m^2^ (or < 21.0 kg/m^2^ if age was ≥ 70 years), weight loss ≥ 5% in the previous month, and/or ≥ 10% in the previous six months. BMI was calculated using the formula weight (kg)/height^2^ (m^2^). Weight was measured within 24 h following admission. Patients were questioned about their current height and their weight one and six months before admission in order to calculate the weight variation. This information was checked in their medical chart when available. Malnutrition was considered as moderate or severe. Severe malnutrition was defined by BMI ≤ 17 kg/m^2^ (or ≤ 18.5 kg/m^2^ if age was ≥ 70 years) and/or weight loss > 10% in the previous month and/or > 15% in the previous six months. Moderate malnutrition was defined by BMI in the range 17.0–18.5 kg/m^2^ (or 18.5–21.0 kg/m^2^ in people ≥ 70 years) and/or weight loss 5 to 10% in the previous month and/or 10 to 15% in the previous six months. Nutritional risk was assessed by calculating the nutritional risk index (NRI): 1.519 x albumin (g/L) + 0.417 × (measured weight/usual weight) × 100 [22]. Malnutrition risk was classified as either no nutritional risk (NRI ≥ 97.5%), moderate nutritional risk (NRI 83.5–97.4%), or severe nutritional risk (NRI < 83.5%). Plasma albumin level was routinely measured at admission according to SFNCM guidelines (see the biological paragraph Section 2.5).

As low albumin levels can result from both inflammation and malnutrition, albumin was adjusted for C reactive protein (CRP) using the following formula: adjusted albumin (g/L) = albumin (g/L) + (CRP (mg/L) − 15)/25. This formula relies on the fact that when CRP is above 15 mg/L, each increase of CRP by 25 mg/L lowers albumin by 1 g/L [23,24]. Accordingly, adjusted NRI was calculated using adjusted albumin instead of measured albumin level.

We explored the causes of malnutrition, including (i) reduced food intake over the last week compared with usual meals; this was evaluated by patient questioning (< 50%, 50–75%, > 75%), (ii) inflammatory disease burden through biomarkers (see below), and (iii) potential reduced food assimilation, such as malabsorption syndromes and diarrhea.

All four COVID-19 departments followed SFNCM therapeutic guidelines [20]. A diagnosis of moderate malnutrition or food intake in the 50–75% range of the usual level for at least one week was signaled to the dietary team, and two oral supplements between meals were added, whereas oral supplementation (polyvitamin, magnesium, phosphorus, vitamin B9, B1, and D) was given for preventing inappropriate refeeding syndrome. When the diagnosis was severe malnutrition or when the food intake was below 50% of the usual level for at least one week, enteral nutrition was started (or at least three oral supplements if respiratory status did not allow enteral nutrition) with intravenous supplementation (phosphorus adapted to its blood level, magnesium, vitamins B1, B9, oligo-elements such as Nutryelt^®^, and polyvitamin including vitamin D such as Cernévit^®^) for preventing inappropriate refeeding syndrome.

### 2.4. Definition of Outcomes

Outcomes were considered during the total length of hospital stay within 28 days of admission. Severe infection was defined as a need for nasal oxygen flow at or above six liters per minute (≥ 6 L/min) during hospitalization (primary outcome). Secondary outcomes included the maximum flow of nasal oxygen reached during hospital stay (with a maximum of 15 L/min), length of stay, need for non-invasive ventilation (if the patient needed more than 15 L/min), transfer to the ICU, and mortality.

We also studied the association between albumin levels (measured at admission) and clinical severity at admission, based on oxygen saturation, respiratory rate, and oxygen pressure on arterial blood gas.

The procedures of care for COVID-19 in our departments were updated as necessary on a daily basis. Basically, antibiotics were initiated when arterial oxygen pressure was below 70 mmHg on blood gas analysis, and larger spectrum antibiotics were used when clinical or biological evolution suggested resistant bacterial infection. Hydroxychloroquine was prescribed in severe respiratory cases at the beginning of the pandemic, when no contraindication was found, but its use was then progressively stopped according to international recommendations and practical observations. Corticosteroids were started with respiratory deterioration at least seven days after the first symptoms, and when inflammation seemed responsible for the critical evolution. Prescription was decided on a multidisciplinary basis.

### 2.5. Data Measurement

Data were anonymously extracted from patients’ charts and collected in a protected health database. We focused on:

General data: patient’s background, usual treatments, the presence or not of current health coverage, or specific social protection including national “universal health coverage” (total coverage of costs granted on a yearly basis to people with low income) and French governmental medical aid to undocumented migrants. We also considered smoking and alcohol habits.

Medical personal history: diabetes, arterial hypertension, and dyslipidemia were self-reported or inferred from the use of, respectively, blood glucose, blood pressure, or lipid-lowering agents. Chronic kidney failure was defined by an estimated glomerular excretion rate below 30 mL/min; cancer and cirrhosis were self-reported or mentioned in the medical charts of the patients.

Ethnicity was reported as Caucasian, Arabic (Middle East, Maghreb), Afro-Caribbean (African, African American, Caribbean), or Asian (Asian continent).

The initial severity of COVID-19 pneumonia was assessed on clinical data (oxygen saturation rate, respiratory frequency), biological data (blood gas under oxygen or ambient air: pH, oxygen arterial pressure (mmHg), and CO2 arterial pressure (mmHg)), and the extent of typical COVID-19 lesions on chest CT scan: mild to moderate (5–25%) vs. critical (> 25%).

The treatments received during hospitalization, including nutritional care (enteral nutrition, oral protein supplements), were reported.

The biomarkers of interest were: (i) nutritional biomarkers: blood proteins (colorimetric analysis), fasting plasma glucose (enzymatic assay with hexokinase/glucose 6 phosphate dehydrogenase, UV test), albumin (immunoturbidimetry), prealbumin (immunoturbidimetry), phosphate (photometric determination of ammonium phosphomolybdate complex), magnesium (colorimetric assay with Chlorophosphonazo III), calcium (colorimetric assay with O-Cresolphtalein), zinc (inductively Coupled Plasma—mass spectrometry), selenium (inductively Coupled Plasma—mass spectrometry), and 25-OH vitamin D (electrochimiluminescence); (ii) inflammation biomarkers: C reactive protein (CRP) (immunoturbidimetry with anti-CRP monoclonal antibodies on latex particles), procalcitonin (fluorometric sandwich immunoassay), ferritin (immunoturbidimetry on latex particles), orosomucoid (immunoturbidimetry, bichromatic assay with two end points at 340 nm and 660 nm), lactate deshydrogenase (kinetics of the conversion of pyruvate into L-lactate), and interleukine 6 (electrochemiluminescence immunoassay). All of these dosages were performed on plasma using a Cobas 6000 analyzer (Roche diagnostics). Finally, fibrinogen (chronometry—Thrombin Dade) was performed on a Siemens analyzer (Siemens).

### 2.6. Statistical Analysis

We estimated for power calculation that 40% of inpatients would suffer from malnutrition [15,25,26,27]. We presumed that the primary outcome (severe pneumonia) would occur in 30% of the malnutrition group of patients vs. 10% of the no malnutrition group. Considering this hypothesis, we needed to include 120 patients to show a significant difference (alpha risk of 5% and power of 80%).

Baseline continuous variables are expressed as mean ± standard deviation. Categorical variables are expressed as frequencies (percentages). We used the Kruskal–Wallis test to compare continuous variables and the chi-squared (X^2^) test or Fisher’s exact test for categorical variables (in a function of the number of patients within each group). All tests were two-sided and used a significance level of *p*-value at 0.05. To assess correlations between two quantitative variables, the non-parametric Spearman coefficients were used. Linear regressions were performed as well, and slopes were tested using Fisher’s test. Logistic regression with the chi-squared test was used for multivariable analyses, including the factors that were associated with COVID-19 severity, with a *p*-value ≤ 0.05 in univariate analyses (as continuous or non-continuous variables) and considering unrelated factors. Additionally, since age was associated with both malnutrition and severe COVID-19 as previously reported [3,4,5,6,7,8], we have adjusted our results for age (chi-squared test with logistic regression). Analyses were conducted with SAS 9.4 software.

### 2.7. Role of the Funding Source

InfuSol and LVL Medical partly funded this study, but they did not participate in any part of the study (collection, analyses, interpretation of data, writing of the report, or decision to submit the report for publication).

## 3. Results

### 3.1. Total Population

A total of 116 patients were admitted between 9 April 2020 and 29 May 2020 in the four dedicated COVID-19 units. Seven patients were excluded because they were not eligible for the ICU in case of aggravation; five had dementia and two had serious comorbidities. We lacked information about nutritional status for one patient. Therefore, 108 consecutive patients were included.

### 3.2. Population Characteristics

Table 1 shows the general and medical characteristics of the total population according to the presence or not of severe COVID-19. The main clinical, biological, and radiological characteristics of patients with or without severe COVID-19 at admission and their treatments are summarized in Table 2. The mean delay between admission and the onset of symptoms was 7.6 ± 5.3 days.

### 3.3. Percentage of Severe COVID-19

A total of 34 patients (31.5%) had severe COVID-19. Fifteen patients (13.9%) needed non-invasive ventilation and fourteen patients (13.0%) were transferred to the ICU. The mean length of stay was 10.5 ± 7.2 days. Five patients (4.6%) died. The maximum flow of nasal oxygen, the length of stay, the need for non-invasive ventilation, the transfer to the ICU, and the mortality rate were greater in the subgroup of patients with severe COVID-19 (Supplementary Table S1).

### 3.4. Percentage and Causes of Malnutrition

Malnutrition was observed in 42 patients (38.9%), with 30 (27.8%) and 12 (11.1%) patients having moderate and severe malnutrition, respectively. The change in food intake could be assessed in 103 patients: food intake was > 75% of its usual level in 20 (19.4%) patients, 50–75% in 25 (24.3%) patients, and < 50% in 58 (56.3%) patients. As shown in Table 3, malnutrition was neither associated with food intake (*p* = 0.06; after adjustment for age, *p* = 0.14) nor with inflammatory biomarkers or digestive symptoms. However, it was associated with age (*p* = 0.04).

Nutritional risk could be assessed in 98 patients, and was observed in 83 patients (84.7%), with 48 (49.0%) and 35 (35.7%) patients having moderate and severe risk, respectively. Nutritional risk was associated neither with food intake nor with digestive symptoms, but was associated with age and with higher levels of several inflammatory biomarkers, including orosomucoid, fibrinogen, CRP, and procalcitonin (Table 3), even after adjustment for age (procalcitonin, *p* = 0.01; orosomucoid, *p* = 0.02; fibrinogen and CRP, *p* < 0.01). Albumin level was associated with the same inflammatory biomarkers, even after adjustment for age (procalcitonin, *p* = 0.01; orosomucoid, fibrinogen, and CRP, *p* < 0.01).

Metabolic biomarkers and nutritional care by nutritional status are presented in Supplementary Table S2. Malnutrition was associated with lower calcium and phosphate levels (*p* = 0.03 and 0.04, respectively). Metabolic parameters, such as plasma protein and albumin and prealbumin levels (*p* < 0.01), were associated with nutritional risk. Patients with nutritional risk received more frequent oral supplements than patients without.

### 3.5. Prognosis Associated with Malnutrition

Malnutrition per se was not associated with severe COVID-19 (Table 4). However, patients with severe COVID-19 were more prone than those with non-severe COVID-19 to have low BMI (*p* = 0.03) and to have lost more weight during the previous month (*p* = 0.05 and 0.08 after adjustment for age) (Table 4). Malnutrition was not associated with maximum nasal oxygen flow (*p* = 0.30) (Supplementary Figure S2), whereas weight loss in one month was associated (*p* < 0.05) (Supplementary Figure S3).

Nutritional risk was positively associated with severe COVID-19 (Table 4). NRI was significantly lower in patients with severe COVID-19, even after adjustment for age (*p* = 0.03). The NRI adjusted for CRP was also associated with severe COVID-19 (*p* < 0.01) (Table 4). Nutritional risk (Supplementary Figure S4, *p* < 0.01), NRI level (Supplementary Figure S5, *p* < 0.01), and NRI level after adjustment for CRP (*p* < 0.05) were associated with maximum nasal oxygen flow. Furthermore, low levels of albumin, prealbumin, protein, and zinc, and higher levels of magnesium, were also associated with the severity of COVID-19 (*p* < 0.01). Albumin adjusted for CRP level was still associated with severe COVID-19 (*p* = 0.03). After adjustment for age, only low levels of protids, zinc, and phosphorus, and high levels of magnesium, were associated with severe COVID-19 (*p* < 0.01) (Table 4).

Albumin level was not associated with initial COVID-19 severity based on oxygen saturation at admission (*p* = 0.06), respiratory rate at admission (*p* = 0.96), and oxygen pressure on arterial blood gas at admission (*p* = 0.83).

### 3.6. Other Parameters Associated With Severe COVID-19

The clinical and personal medical history parameters significantly associated with severe COVID-19 were older age (Table 1), lower saturation in ambient air, and higher respiratory rate at admission (Table 2). Patients who experienced severe COVID-19 were more prone to have a positive PCR for SARS-CoV-2. Additionally, ferritin and orosomucoid levels were higher in patients with severe COVID-19 (Table 2).

Logistic regression that integrated age, PCR result, ferritin, low BMI, and NRI showed that older age (odds ratio (OR) per 10 years = 1.69 (95% confidence interval, 1.08–2.65), *p* = 0.02), positive PCR (OR = 4.85 (1.08–21.74), *p* = 0.04), and higher ferritin level (OR per 1000 µg/L = 3.15 (1.52–6.50), *p* = 0.02) were independently associated with severe COVID-19 (Supplementary Table S3). The same logistic regression including BMI as a continuous variable instead of low BMI showed similar results (Supplementary Table S3). A third model integrating age, ferritin, and NRI by classes, as well as PCR result and low BMI, showed that positive PCR (OR = 7.77 (1.58–37.85), *p* = 0.01) and ferritin level > 600 µg/L (OR = 20 (3–131), *p* = 0.001) were independently associated with severe COVID-19 (Supplementary Table S3).

### 3.7. Secondary Outcomes

Supplementary Table S4 shows that secondary outcomes were not associated with malnutrition. Maximum nasal oxygen flow was associated with nutritional risk (*p* < 0.01).

## 4. Discussion

Our study has shown that malnutrition was highly prevalent in patients admitted in departments dedicated to COVID-19. Malnutrition, based on low BMI and/or weight loss, was present in 38.9% of patients, whereas nutritional risk, based on albumin level and weight loss, was moderate in 49.0% and severe in 35.7% of patients. Furthermore, we report that nutritional risk was associated with severe COVID-19.

The French definition of malnutrition we used is similar to the one of the Global Leadership Initiative on Malnutrition (GLIM), and relies on the same phenotypic and etiologic criteria, except for the edges of BMI, which are higher in the GLIM definition, therefore underestimating malnutrition in our cohort [26]. The prevalence of malnutrition in hospitalized patients has been reported to be 27 to 55% regardless of the cause of hospitalization [25,28,29]. Specifically, Yeo et al. reported a prevalence of 39% in a cohort of 198 Korean patients admitted for community-acquired pneumonia [15], as in our series. Recently, malnutrition was found in 52.7% of elderly COVID-19 inpatients using the Mini Nutritional Assessment (MNA) [2]. Moreover, based on the modified NUTRIC score at ICU admission, a high nutritional risk (≥ 5 points) was observed in 61% of the critically ill COVID-19 patients [27]. In our study, nutritional risk might be overestimated by low albumin levels through inflammation, as discussed below.

We explored the causes of malnutrition. According to the GLIM conference [26], disease- or injury-related malnutrition is due to a combination of reduced food intake or assimilation and varying degrees of acute or chronic inflammation. Indeed, food intake was largely reduced in our population with COVID-19: the reduction in food intake over the last week was more than 50% for 56.3% of the patients. Considering the mean delay between the first symptoms and admission of approximately a week, this observation may suggest that nutrition support should be provided before admission to every COVID-19 patient. Reduced food intake may partly rely on gustative dysfunction and digestive symptoms experienced in some patients with COVID-19, but there was no significant association of these symptoms with malnutrition. Inflammation contributes to malnutrition through anorexia and decreased food intake. It alters metabolism by elevating and increasing muscle catabolism [30]. Finally, only a few patients had severe underlying comorbidities exposing to chronic malnutrition, meaning that malnutrition in COVID-19 results from an acute event.

Even though we could not show a significant association between malnutrition and COVID-19 severity, low BMI and weight loss considered as a continuous variable were associated with severe infection. In the recent cohort of critically ill patients in Wuhan, the mortality of ICU within 28 days was significantly higher in the high nutritional risk group than in the low nutritional risk group (87 vs. 49%, *p* < 0.001). Patients in the high nutritional risk group exhibited significantly higher incidences of acute respiratory distress syndrome, acute myocardial injury, secondary infection, shock, and use of vasopressors. Additionally, use of a multivariate Cox analysis showed that patients with high nutritional risk had a higher probability of death at 28 days in the ICU than those with low nutritional risk (adjusted HR = 2.01, 95% CI: 1.22–3.32, *p* = 0.006) [27]. Furthermore, in a review of 25 death cases, malnutrition was common in severe patients [31]. In the Korean study by Yeo et al., malnutrition was associated with a two-fold increase in two-year mortality [15]. Nevertheless, the authors did not observe any significant difference in short-term outcomes according to the nutritional status. Looking at influenza infections, malnutrition was identified as a strong and independent predictor of mortality [32].

In a recent study, the NRI was considered as a useful and practical tool for the screening of patients with COVID-19 needing additional nutritional intervention [22]. In addition, our results suggest that this simple tool could be used in any country as a predictor of severity, and could participate in guiding nutritional management. Indeed, the NRI was significantly and proportionally associated with severe COVID-19 in our series, even after adjustment for age.

Nutritional risk is a combination of hypoalbuminemia and weight loss, which are the results of the combined effects of inflammation and inadequate protein and caloric intake. Inflammation induces anorexia, reduces the effective use of dietary protein and energy intake, and augments catabolism of the key somatic protein, albumin [33]. In a large cohort of 2645 patients from the emergency room, elevated parameters of inflammation and high nutritional risk were independently associated with hypoalbuminemia, but all three parameters independently predicted mortality [34]. Therefore, inflammation could fully explain both an increased NRI and a poor prognosis. However, the NRI adjusted for CRP level remained associated with severe COVID-19, meaning that neither inflammation nor age entirely explained the association. In addition, albumin levels were not associated with severe COVID-19 at admission. This suggests that a low albumin level, and therefore low NRI, might not reflect current but future severity. On the contrary and in agreement with previous COVID-19 studies [3,4,5,6,7,8], we found an independent association between age, initial infection presentation, inflammatory biomarkers, and infection severity, suggesting that the poor prognosis associated with nutritional risk is partly driven by inflammation [30]. Therefore, we think that nutritional risk and malnutrition are partly a consequence of COVID-19, but might also expose to a higher risk of severe disease and help identify the patients who need particular attention.

Several nutritional biomarkers were associated with pneumonia’s prognosis. Albumin [5] and pre-albumin [7] levels have already been described as prognostic factors for COVID-19 severity. Very recently, albumin has been shown to be a predictor of mortality [35]. Zinc is known to modulate antiviral and antibacterial immunity and to regulate inflammatory response [36]. Furthermore, some studies suggest that zinc supplementation may be of benefit for the prophylaxis and treatment of COVID-19 [37]. Magnesium level was surprisingly higher in patients with severe COVID-19. This might reflect magnesium supplementation in the most severe patients, but this is unlikely, as biomarkers were measured at admission.

As we explored nutritional status right after admission, our results suggest that malnutrition is present in the early stage of COVID-19 and should be considered when symptoms begin. These results support the European Society for Clinical Nutrition and Metabolism (ESPEN) guidelines for appropriate nutritional assessment and treatment, as it is well-documented to effectively reduce complications and improve relevant clinical outcomes under various conditions, including ICU stays, hospitalization, and several chronic diseases [38].

Indeed, a recent review has shown the importance of maintaining a correct nutritional status of 10 nutrients analyzed for the health of the immune system, highlighting especially the importance of Vitamin D and iron in the context of COVID-19 [39,40,41]. A wealth of mechanistic and clinical data has shown that vitamins, including vitamins A, B_6_, B_12_, C, D, E, and folate; trace elements, including zinc, iron, selenium, magnesium, and copper; and the omega-3 fatty acids eicosapentaenoic acid and docosahexaenoic acid play important and complementary roles in supporting the immune system. Inadequate intake and status of these nutrients are widespread, leading to a decrease in resistance to infections and, as a consequence, an increase in disease burden [42]. Another recent review suggests a potential interventional role of nutrients to strengthen the immune system against the emerging infection caused by COVID-19 [43].

A limitation of our study is that we did not include 120 patients as recommended considering power calculation. Actually, the study started when our local nutrition committee gave written recommendations on nutritional care, giving the opportunity to routinely assess nutritional phenotype and biomarkers in all consecutive patients. At this time, the COVID-19 pandemic thankfully decreased in our region. Nevertheless, we could include 108 patients, and this number was sufficient to show an association between low BMI, weight loss, NRI, nutritional biomarkers, and COVID-19 severity. Our population was not representative of all COVID-19 patients, as we only investigated patients admitted for acute infection. Therefore, information only applies to critically ill patients, with symptoms too severe to stay at home because of either respiratory symptoms or severe comorbidities, but not severe enough to be transferred into the ICU or to directly require tracheal intubation. This population looked more prone to malnutrition than ambulatory patients who were less likely at risk for malnutrition and severe pneumonia. On the other hand, ICU patients usually have malnutrition independently of the disease [44], and could not be questioned about their weight loss.

The strengths of our study include a multiethnic cohort likely to be translatable for different populations and a pragmatic guidance-based approach. The main outcome (need for oxygen ≥ 6 L/min) was accurate for evaluating pneumonia’s severity, as it was strongly correlated to length of stay, non-invasive ventilation, transfer into the ICU, and mortality. Finally, despite the rapid pace of emerging scientific information during this pandemic, the ASPEN scoping review has identified multiple critical areas for urgent nutrition research [45], making this research topic interesting to us.

## 5. Conclusions

To conclude, in our population, about 40% of patients admitted for COVID-19 pneumonia had malnutrition and about 35% of them had severe nutritional risk. Food intake was usually reduced in infected patients. Nutritional risk was associated with severe COVID-19, probably both as a cause and a consequence. Our results support guidelines for the screening of patients at risk of malnutrition and providing nutritional support for those patients [1,38]. However, only randomized controlled studies could demonstrate that such guidance could improve COVID-19 prognosis in critically ill patients outside of the ICU, and such a study would appear unethical, as nutritional support is harmless and improving COVID-19 management is an emergency.

## Figures and Tables

**Table 1:** Population characteristics at admission.

	N	Total	Non severe COVID-19	Severe COVID-19	*p*
Number of patients		n = 108	n = 74	n = 34	
Age (years)	108	61.8 ± 15.8	58.9 ± 15.2	68.0 ± 15.4	<0.01
Male gender	108	64 (59.3)	40 (51.1)	24 (70.6)	0.10
Body mass index (kg/m^2^)	108	28.8 ± 6.2	29.0 ± 5.85	28.2 ± 7.0	0.84
Ethnicity	107				0.21
Caucasian		35 (32.7)	22 (29.7)	13 (39.4)	
Arabic		35 (32.7)	22 (29.7)	13 (39.4)	
Afro-Caribbean		20 (18.7)	15 (20.3)	5 (15.2)	
Asian		17 (15.9)	15 (20.3)	2 (6.1)	
Health coverage	98				0.06
Current health coverage		75 (76.5)	49 (70.0)	26 (92.9)	
Specific social protection		18 (18.4)	16 (22.9)	2 (7.1)	
No health coverage		5 (5.1)	5 (7.1)	0 (0.0)	
No smoking	99	97 (98.0)	68 (97.1)	29 (100)	>0.99
No alcohol consumption	100	93 (93.0)	66 (93.0)	27 (93.1)	>0.99
Personal history					
Diabetes	108	45 (41.7)	29 (39.2)	16 (47.1)	0.44
Arterial hypertension	108	60 (55.6)	39 (52.7)	21 (61.8)	0.38
Dyslipidemia	105	35 (33.3)	22 (30.6)	13 (39.4)	0.37

Continuous variables are expressed as mean ± standard deviation. Categorical variables are expressed as the number of patients (percentage). N: number of available data. A severe infection was defined as a need for nasal oxygen flow of at or above 6 L per minute. COVID-19: Sars-Cov-2 infectious disease.

**Table 2:** Clinical, biological, and radiological data at admission and COVID-19 treatments.

	N	Total	Non Severe COVID-19	Severe COVID-19	*p*
Number of patients		n = 108	n = 74	n = 34	
Pulmonary extent of infection on chest CT	96				0.06
Mild to moderate		74 (77.1)	53 (82.8)	21 (65.6)	
Extended to critical		22 (22.9)	11 (17.2)	11 (34.4)	
Positive Sars-Cov-2 PCR	105	76 (72.4)	46 (63.9)	30 (90.9)	<0.01
Initial ambient air saturation (%)	104	94.3 ± 4.1	95.4 ± 2.8	91.7 ± 5.2	<0.01
Respiratory rate (/min) at admission	95	26.7 ± 6.4	25.5 ± 6.3	29.2 ± 6.0	< 0.01
Symptoms at admission					
Respiratory	108	85 (78.7)	55 (74.3)	30 (88.2)	0.10
Diarrhea	107	19 (17.8)	16 (21.6)	3 (9.1)	0.12
Nausea/vomiting	108	16 (14.8)	12 (16.2)	4 (11.8)	0.55
Neurological symptoms (except headache)	108	13 (12.0)	8 (10.8)	5 (14.7)	0.54
Olfactory/gustatory dysfunction	107	13 (12.1)	6 (8.2)	7 (20.6)	0.11
Inflammation markers					
Ferritin (µg/L)	95	1070 ± 2069	676 ± 696	1846 ± 3328	<0.01
Orosomucoid (g/L)	52	1.9 ± 0.6	1.7 ± 0.7	2.1 ± 0.5	0.05
Fibrinogen (g/L)	103	5.2 ± 1.5	5.1 ± 1.6	5.6 ± 1.4	0.08
C-reactive protein (mg/L)	108	78 ± 85	72 ± 82	91 ± 91	0.29
Procalcitonin (µg/L)	105	0.7 ± 2.9	0.8 ± 3.2	0.6 ± 2.2	0.17
Interleukin 6 (pg/mL)	43	64.3 ± 71.2	44.9 ± 43.9	82.9 ± 86.9	0.30
Lactate dehydrogenase (U/L)	42	379 ± 230	336 ± 176	486 ± 313	0.07
COVID-19 treatment					
Antibiotics	108	91 (84.3)	57 (77.0)	34 (100)	<0.01
Change in antibiotics	108	12 (11.1)	1 (1.4)	11 (32.4)	<0.01
Hydroxychloroquine	103	10 (9.7)	3 (4.2)	7 (21.9)	<0.01
Corticosteroids	105	28 (26.7)	8 (11.3)	20 (58.8)	<0.01

Continuous variables are expressed as mean ± standard deviation. Categorical variables are expressed as the number of patients (percentage). N: number of available data. COVID-19: Sars-Cov-2 infectious disease; CT: computed tomography; PCR: polymerase chain reaction. Severe COVID-19 was defined as a need for nasal oxygen flow at or above 6 L per minute.

**Table 3:** Causes of malnutrition and nutritional risk.

	n	No Malnutrition	Malnutrition *	*p*-Value	n	No Nutritional Risk	Nutritional Risk **	*p*-Value
Number of patients	108	n = 66	n = 42		98	n = 15	n = 83	
Age (years)	108	59.0 (15.6)	66.1 (15.3)	0.04		50.1 (15.9)	62.7 (14.9)	<0.01
Food intake	103			0.06	83			0.59
< 50%		30 (47.6)	28 (70.0)			7 (46.7)	46 (59.0)	
50–75%		17 (27.0)	8 (20.0)			4 (26.7)	18 (23.1)	
> 75%		16 (25.4)	4 (10.0)			4 (26.7)	14 (17.9)	
Inflammation biomarkers								
Ferritin (µg/L)	95	861 ± 849	1429 ± 3221	0.38	92	538 ± 460	1191 ± 2244	0.11
Orosomucoid (g/L)	52	1.8 ± 0.6	1.9 ± 0.6	0.84	48	1.3 ± 0.5	2.0 ± 0.6	<0.01
Fibrinogen (g/L)	103	5.1 ± 1.7	5.4 ± 1.3	0.30	94	3.8 ± 1.4	5.5 ± 1.5	<0.01
C-reactive protein (mg/L)	108	75 ± 88	82 ± 79	0.30	98	17 ± 24	90 ± 90	<0.01
Procalcitonin (µg/L)	105	0.5 ± 2.9	1.0 ± 2.8	0.14	97	0.1 ± 0.1	0.9 ± 3.3	<0.01
Interleukine 6 (pg/mL)	43	61 ± 81	68 ± 57	0.35	43	72 ± 135	63 ± 61	0.22
Lactate dehydrogenase (U/L)	42	397 ± 245	352 ± 211	0.40	37	512 ± 475	363 ± 172	0.46
Symptoms								
Respiratory	108	53 (80.3)	32 (76.2)	0.61	98	13 (86.7)	66 (79.5)	0.73
Diarrhea	107	11 (16.9)	8 (19.0)	0.78	97	5 (33.3)	13 (15.9)	0.15
Nausea/vomiting	108	8 (12.1)	8 (19.0)	0.32	98	3 (20.0)	13 (15.7)	0.71
Neurology (except headache)	108	10 (15.2)	3 (7.1)	0.21	98	3 (20.0)	7 (8.4)	0.18
Olfactory/gustatory dysfunction	107	8 (12.1)	5 (12.2)	>0.99	97	1 (6.7)	12 (14.6)	0.68
Background								
Chronic kidney failure	108	2 (3.1)	2 (4.9)	0.64	98	0 (0.0)	3 (3.6)	>0.99
Cancer	108	4 (6.1)	5 (11.9)	0.31	98	0 (0.0)	8 (9.6)	0.21

Continuous variables are expressed as mean ± standard deviation and categorical variables as the number of patients (percentage). N: number of available data. * Malnutrition was defined as body mass index < 18.5 kg/m^2^ (or < 21 kg/m^2^ if age was ≥ 70 years), weight loss ≥ 5% in one month, and/or weight loss ≥ 10% in six months. ** Nutritional risk was defined as nutritional risk index (1.519 × albumin (g/L) + 0.417 × (measured weight/usual weight) × 100) < 97.5.

**Table 4:** Prognosis of malnutrition and nutritional risk.

	N	Total	Non Severe COVID-19	Severe COVID-19	*p*-Value	*p*-Value After Adjustment for Age
Number of patients		n = 108	n = 74	n = 34		
Malnutrition						
Presence of malnutrition	108	42 (38.9)	25 (33.8)	17 (50.0)	0.11	0.30
Malnutrition:	108				0.19	0.46
Absent		66 (61.1)	49 (66.2)	17 (50.0)		
Moderate		30 (27.8)	19 (25.7)	11 (32.4)		
Severe		12 (11.1)	6 (8.1)	6 (17.6)		
Low body mass index *	108	5 (4.7)	1 (1.4)	4 (11.8)	0.03	
Weight loss within one month (kg)	108	3.7 ± 6.8	2.6 ± 5.9	6.1 ± 8.1	0.05	0.08
Weight loss ≥ 5% within one month	108	40 (37.0)	25 (33.8)	15 (44.1)	0.30	0.54
Weight loss ≥ 10% within six months	96	10 (10.4)	6 (9.0)	4 (13.8)	0.48	0.84
Nutritional risk						
Nutritional risk index (%) **	98	87 ± 9	89 ± 9	83 ± 8	<0.01	0.03
Adjusted nutritional risk index (%)	98	91 ± 8.4	92 ± 8.3	88 ± 7.9	<0.01	0.13
Nutritional risk status	98				0.01	0.05
Absence		15 (15.3)	13 (20.0)	2 (6.1)		
Moderate		48 (49.0)	35 (53.8)	13 (39.4)		
Severe		35 (35.7)	17 (26.2)	18 (54.5)		
Biological markers						
Albumin (g/L)	98	30.9 ± 5.6	31.9 ± 5.5	29.0 ± 5.4	<0.01	0.10
Adjusted albumin	98	33.5 ± 5.2	34.2 ± 4.7	32.1 ± 5.9	0.03	0.10
Prealbumin (g/L)	89	0.15 ± 0.07	0.16 ± 0.06	0.13 ± 0.08	<0.01	0.20
Plasma proteins (g/L)	108	67.2 ± 9.2	69.6 ± 8.3	61.8 ± 8.8	<0.01	<0.01
Zinc (mg/L)	47	0.7 ± 0.2	0.7 ± 0.2	0.6 ± 0.1	<0.01	<0.01
Selenium (µg/L)	53	82.4 ± 18.7	85.9 ± 17.7	76.3 ± 19.4	0.12	0.18
Calcium (mmol/L)	100	2.18 ± 0.15	2.19 ± 0.15	2.15 ± 0.15	0.34	0.39
Phosphorus (mmol/L)	100	1.03 ± 0.22	1.06 ± 0.22	0.99 ± 0.21	0.10	<0.01
Magnesium (mmol/L)	95	0.83 ± 0.11	0.80 ± 0.11	0.87 ± 0.12	<0.01	<0.01
25-OH vitamin D (ng/mL)	67	15.4 ± 12.5	15.9 ± 13.8	14.3 ± 10.1	0.88	0.49
Fasting plasma glucose (mmol/L)	104	7.1 ± 3.0	6.9 ± 2.6	7.5 ± 3.6	0.33	0.23
Nutritional management						
Oral supplements	108	69 (63.9)	41 (55.4)	28 (82.4)	<0.01	0.04
Enteral nutrition	108	3 (2.8)	0 (0.0)	3 (8.8)	0.03	0.98
Vitamin supplementation	107	37 (34.6)	19 (26.0)	18 (52.9)	<0.01	0.02

Continuous variables are expressed as mean ± standard deviation. Categorical variables are expressed as the number of patients (percentage). N: number of available data. * A low body mass index was defined as ≤ 18.5 kg/m^2^ (or ≤ 21 kg/m^2^ if age was ≥ 70 years). Severe COVID-19 was defined as a need for nasal oxygen flow at or above 6 L per minute. ** Nutritional risk index (NRI): 1.519 × albumin (g/L) + 0.417 × (measured weight/usual weight) × 100. Nutritional risk according to NRI is no malnutrition (NRI ≥ 97.5%), moderate malnutrition (NRI 83.5–97.4%), or severe malnutrition (NRI < 83.5%).

## Supplementary Materials

The following are available online at https://www.mdpi.com/2072-6643/12/12/3679/s1, Figure S1: Flow Chart, Figure S2: maximal nasal oxygen flow according to clinical malnutrition, Figure S3: maximal nasal oxygen flow according to weight loss in one month, Figure S4: maximal nasal oxygen according to nutritional risk, Figure S5: maximal nasal oxygen by nutritional risk index, Table S1: Correlation between secondary outcomes and severe COVID-19; Table S2: Nutritional biomarkers and care according to nutritional status, Table S3: Multivariable analysis explaining COVID-19 severity, Table S4: Secondary outcomes according to nutritional status.
