# Supplementary material for: Malnutrition: Percentage and Association with Prognosis in Patients Hospitalized for Coronavirus Disease 2019
Source: Nutrients. 2020 Nov 28;12(12):3679. doi: 10.3390/nu12123679 (PMC7761464; doi:10.3390/nu12123679)
Supplement: Supplementary file 1 [file nutrients-12-03679-s001.pdf]

Prevalence of malnutrition and its consequences on prognosis in patients hospitalized for SARS-Cov2 virus infection

First Author: Lucie Allard

Online supplementary material

Supplementary figure 1: Flow Chart

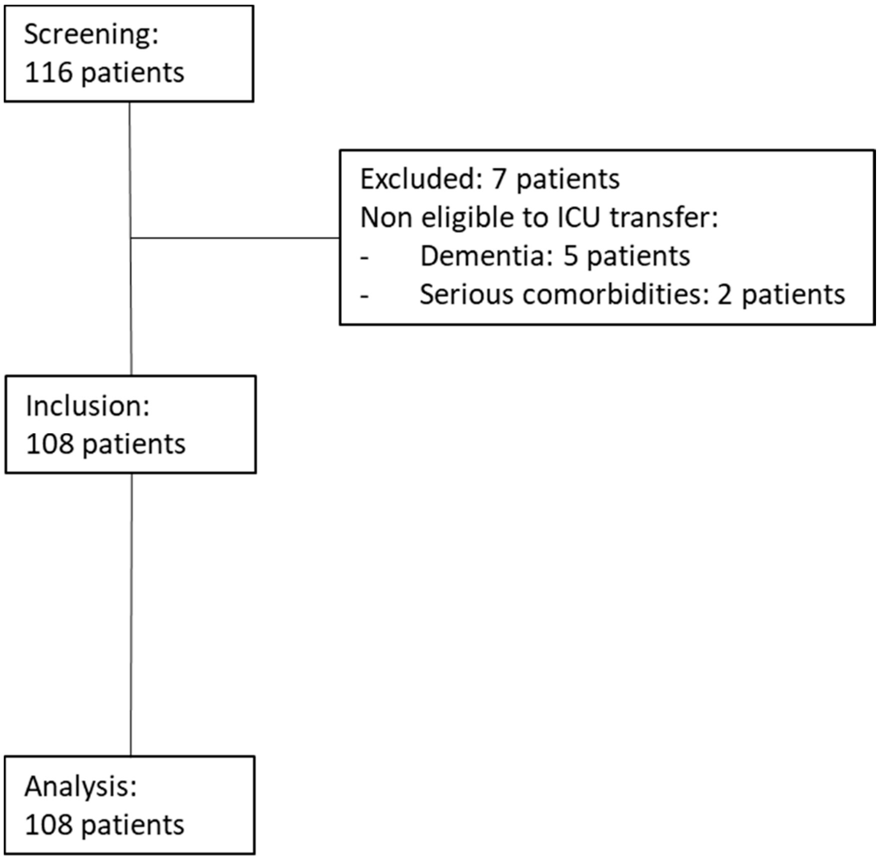

ICU: intensive care unit

All patients included were analyzed

Supplementary Figure 2: maximal nasal oxygen flow according to clinical malnutrition

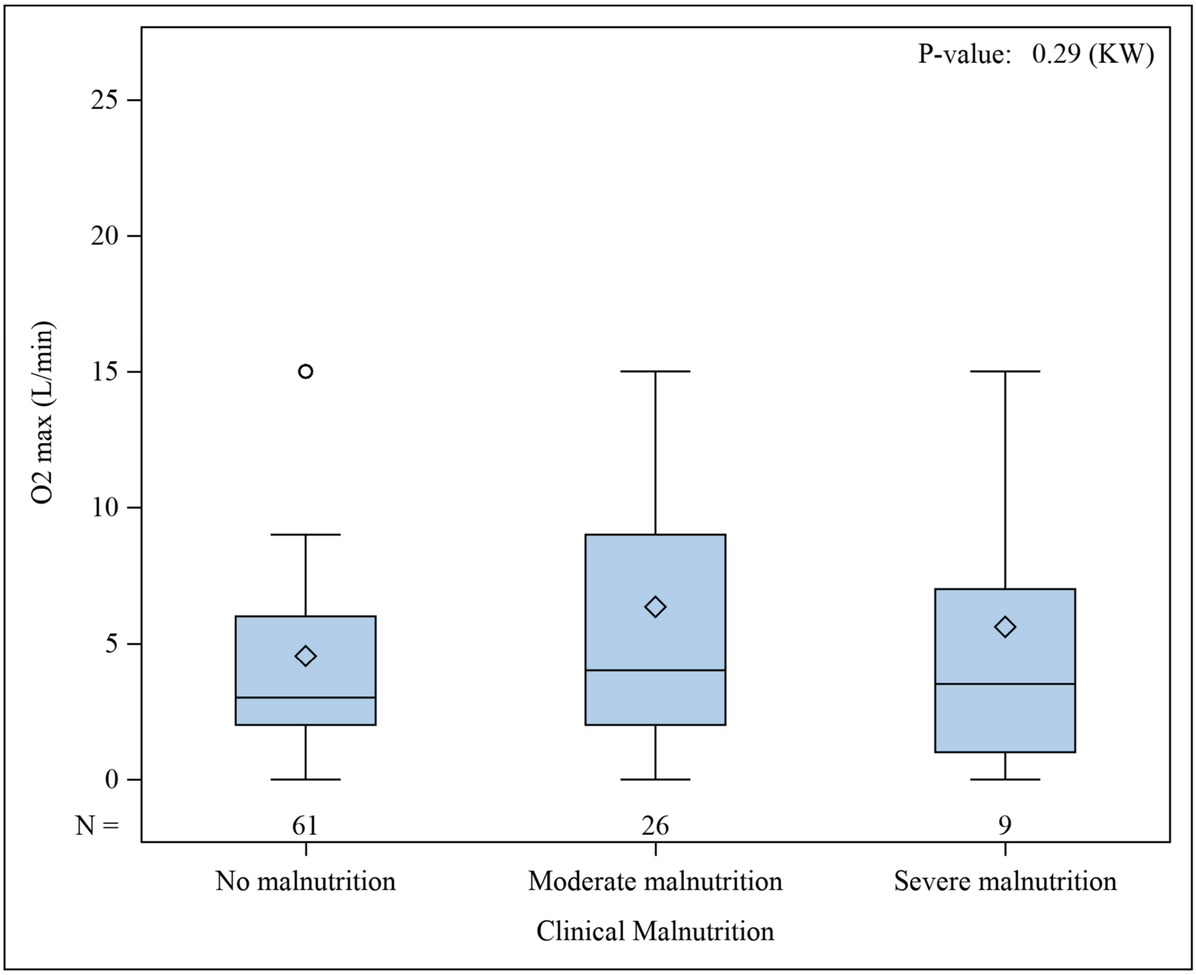

Clinical malnutrition: body mass index (BMI) < 18·5 kg/m² (or < 21 kg/m² if age was ≥ 70 years) or weight loss ≥ 5% in one month, and/or weight loss ≥ 10% in six months

Severe clinical malnutrition: BMI ≤ 17·0 kg/m² (or ≤ 18·5 kg/m² if age was ≥ 70 years) and/or weight loss > 10% in 1 month and/or > 15% in 6 months

Supplementary Figure 3: maximal nasal oxygen flow according to weight loss in one month

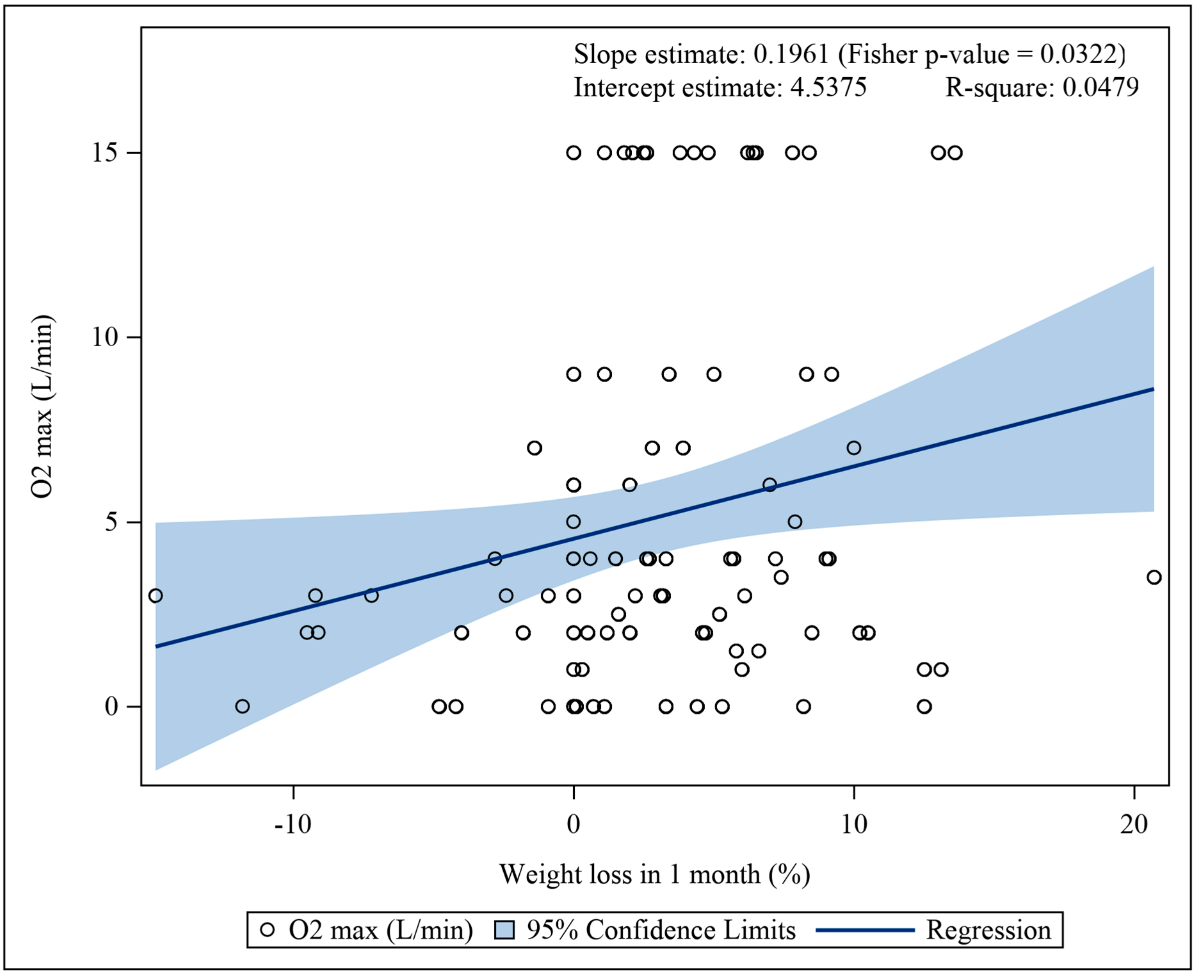

Supplementary Figure 4: maximal nasal oxygen according to nutritional risk

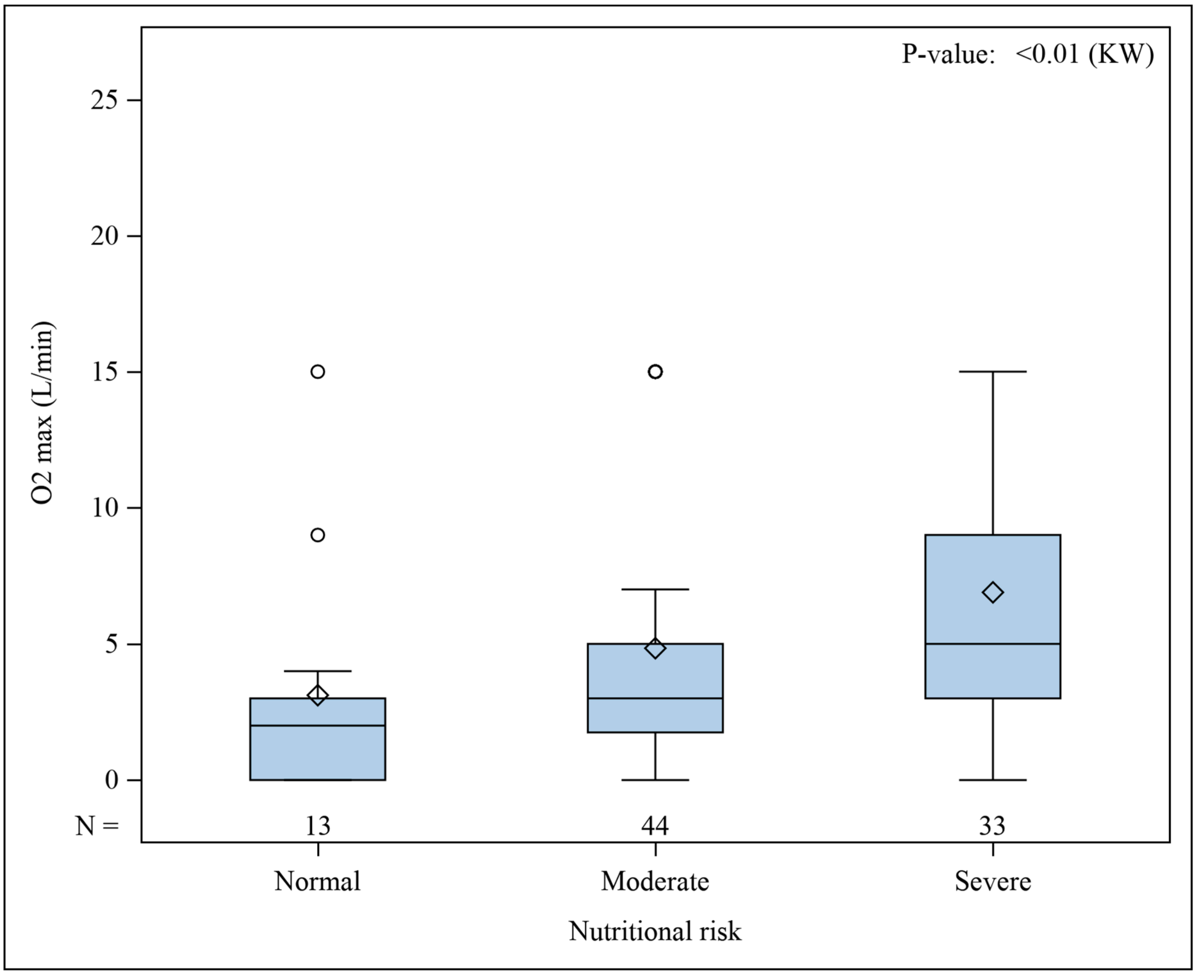

Nutritional risk index (NRI): 1·519 x albumin (g/l) + 0·417 x (measured weight/ usual weight) x 100. Nutritional risk status is defined according to NRI level: no nutritional risk (NRI ≥ 97·5%), moderate nutritional risk (NRI 83·5-97·4%) and severe nutritional risk (NRI < 83·5%).

Supplementary Figure 5: maximal nasal oxygen by nutritional risk index

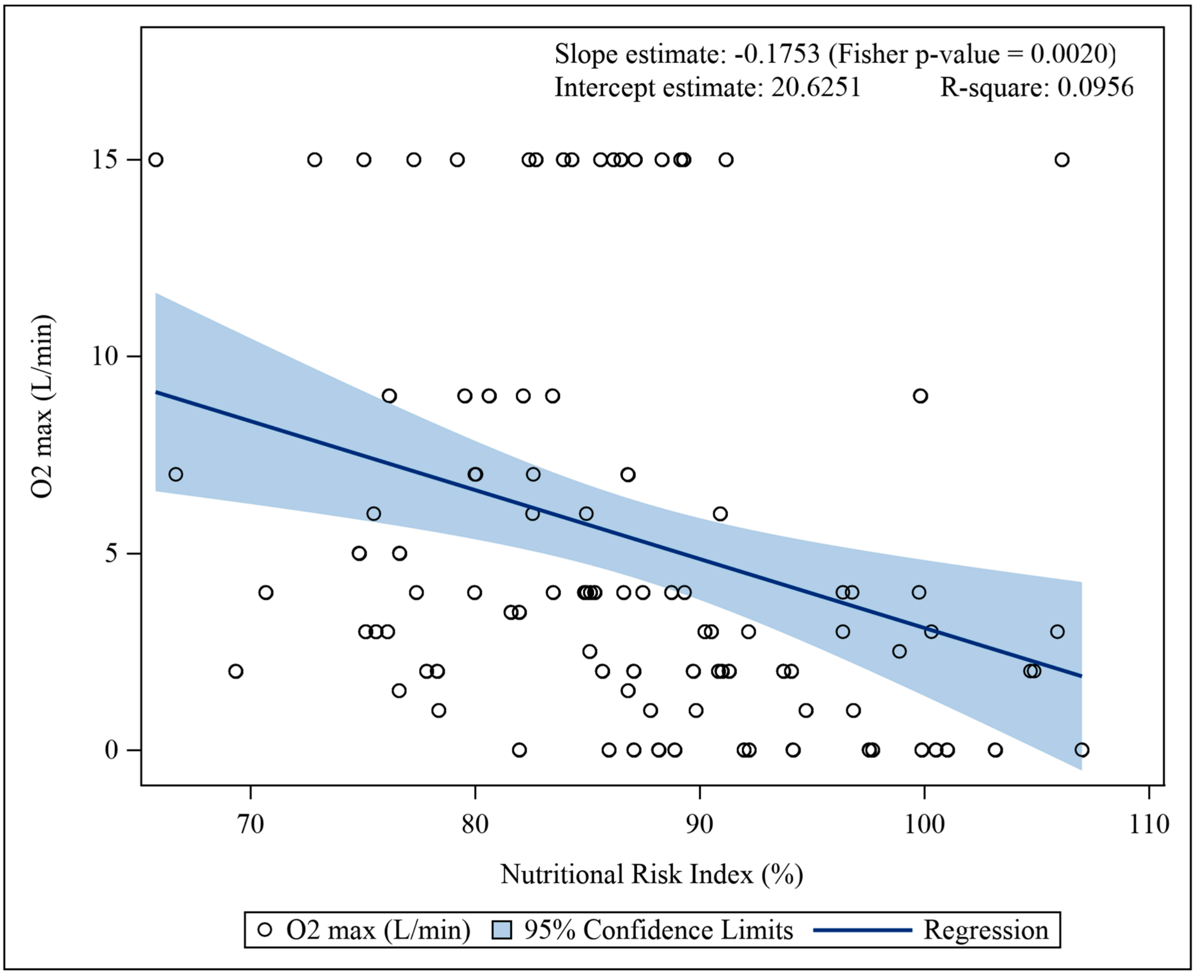

Nutritional risk index is calculated as 1·519 x albumin (g/l) + 0·417 x (measured weight / usual weight) x 100.

Supplementary Table 1: Correlation between secondary outcomes and severe COVID-19

	N	Total	Non severe COVID-19	Severe COVID-19	p
		n = 108	n = 74	n = 34	
Maximum flow of nasal O2 (L/min)	108	4·8 ± 5.0	2·1 ± 1·6	10·7 ± 4·9	<0·01
Lengh of stay (days)	98	10·5 ± 7·2	8.6 ± 5·9	15·7 ± 7·9	<0·01
Non invasive ventilation	108	15 (13·9)	0 (0·0)	15 (44·1)	<0·01
Transfer into the intensive care unit	108	14 (13·0)	3 (4·1)	11 (32·4)	<0·01
Mortality	108	5 (4·6)	0 (0·0)	5 (14·7)	<0·01

Continuous variables are expressed as mean ± standard deviation. Categorical variables are expressed as number of patient (percentage). N: number of available data

COVID-19: Coronavirus disease 2019. Severe COVID-19 was defined as a need for nasal oxygen flow at or above six liters per minute.

Supplementary Table 2: Nutritional biomarkers and care according to nutritional status

	n	No clinical malnutrition	Clinical malnutrition*	p	n	No nutritional risk	Nutritional risk**	p
	108	n=66	n=42		98	n=15	n=83	
**Nutritional biomarkers**								
Albumin (g/l)	98	31·3 ± 5·8	30·3 ± 5·1	0·45	98	39·6 ± 2·1	29·3 ± 4·4	<0·01
Prealbumin (g/l)	89	0·16 ± 0·07	0·13 ± 0·06	0·11	87	0·14 ± 0·06	0·23 ± 0·08	<0·01
Protid (g/l)	108	68·2 ± 10·3	65·6 ± 6·9	0·17	98	75·3 ±5·5	65·9 ± 9·1	<0·01
Zinc (mg/l)	47	0·7 ± 0·2	0·7 ± 0·2	0·80	44	0·7 ± 0·2	0·7 ± 0·2	0·73
Selenium (µg/l)	53	85·8 ± 18·7	77·7 ±18·1	0·15	50	83·2 ± 7·5	81·8 ± 18·2	0·85
Calcium (mmol/l)	100	2·2 ± 0·1	2·1 ± 0·1	0·03	92	2·4 ± 0·1	2·1 ± 0·1	<0·01
Phosphore (mmol/l)	100	1·1 ± 0·2	1·0 ± 0·2	0·04	92	1·1 ± 0·3	1·0 ± 0·2	0·18
Magnesium (mmol/l)	95	0·8 ± 0·1	0·8 ± 0·1	0·28	88	0·8 ± 0·1	0·8 ± 0·1	0·80
25-OH vitamin D (ng/ml)	67	14·3 ± 11·5	17·4 ± 14·4	0·23	64	11·4 ± 3·9	15·9 ± 13·1	0·88
Fasting plasma glucose (mmol/l)	104	6·8 ± 2·9	7·6 ± 3·0	0·08	95	6·9 ± 2·3	7·2 ± 3·1	0·99
**Nutritional care**								
Oral supplements	108	38 (57·6)	31 (73·8)	0·09	98	3 (20·0)	62 (74·7)	<0·01
Enteral nutrition	108	0 (0·0)	3 (7·1)	0·06	98	0 (0·0)	3 (3·6)	1·00
Vitamin supplementation	107	22 (33·8)	15 (35·7)	0·84	97	4 (26·7)	32 (39·0)	0·36

* Clinical malnutrition: body mass index (BMI) < 18·5 kg/m² (or < 21 kg/m² if age was ≥ 70 years) or weight loss ≥ 5% in one month, and/or weight loss ≥ 10% in six months

** Nutritional risk is defined as a Nutritional Risk Index (NRI) < 97·5. NRI = 1·519 x albumin (g/l) + 0·417 x (measured weight/ usual weight) x 100.

Supplementary Table 3: Multivariable analysis explaining COVID-19 severity

	Odds Ratios [95% confidence interval]	p
Age (by 10 years)	1.687 [1.076- 2.647]	0.023
PCR for SARS-CoV-2 (ref = positive)	4.852 [1.083- 21.737]	0.039
Ferritin (by 1000 µg/l))	3.149 [1.525- 6.501]	0.002
Low body mass index* (ref = No)	0.847 [0.056-12.864]	0.90
Nutritional Risk Index (by 10 units)	0.650 [0.318-1.329]	0.24
	**Odds Ratios [95% confidence interval]**	**p**
Age (by 10 units)	1.687 [1.086 ; 2.621]	0.0199
PCR for SARS-CoV-2 (ref = No)	4.766 [1.064 ; 21.353]	0.0413
Ferritin (by 1000 units)	3.135 [1.517 ; 6.477]	0.0020
BMI (by 1 unit)	1.008 [0.917 ; 1.109]	0.8632
Nutritional Risk Index (by 10 units)	0.653 [0.324 ; 1.315]	0.2329
	**Odds Ratios [95% confidence interval]**	**p**
Age 51-60 y.o. vs <= 50 y.o.	2.644 [0.310 ; 22.538]	0.1358
Age 61-75 y.o. vs <= 50 y.o.	6.754 [1.164 ; 39.178]	0.1358
Age > 75 y.o. vs <= 50 y.o.	8.310 [1.014 ; 68.098]	0.1358
PCR COVID (ref = No)	7.775 [1.597 ; 37.855]	0.0111
Ferritin 301-600 vs <= 300	1.219 [0.090 ; 16.568]	0.0015
Ferritin 601-1000 y.o. vs <= 300	19.965 [3.053 ; 130.550]	0.0015
Ferritin >1000 vs <= 300	20.932 [3.121 ; 140.387]	0.0015
Low BMI (ref = No)	3.797 [0.126 ; 114.259]	0.4424
Nutritional Risk Index Moderate (ref = normal)	0.740 [0.086 ; 6.357]	0.6734
Nutritional Risk Index Severe (ref = normal)	1.327 [0.151 ; 11.658]	0.6734

*A low body mass index was defined as ≤ 18·5 kg/m² (or ≤ 21 kg/m² if age was ≥ 70 years).

Ref: reference; PCR: polymerase chain reaction

Logistic regression was used for multivariable analysis based on models including the factors (considered as continuous or non continuous) that were associated with COVID-19 severity with a p value ≤ .05 in univariate analyses and considering unrelated factors. Therefore, we considered (i) Sars Cov2 PCR status but not initial ambient air saturation nor respiratory rate at admission, (ii) ferritin (n=95) rather than orosomucoid (n=52), and (iii) NRI and not albumin and weight loss in one month which are part of its definition. We did not include prealbumin nor plasma proteins which were highly related to NRI; neither Zinc level (n=47).

Supplementary Table 4: Secondary outcomes according to nutritional status

	n	No clinical malnutrition	Clinical malnutrition*	p	n	No nutritional risk	Nutritional risk**	p
	108	n=66	n=42		98	n=15	n=83	
Maximum flow of nasal oxygen (L/min)	108	4·5 ±4.8	6·0 ±5·3	0·12	98	2·7 ±4·2	5·8 ±5·2	<0·01
Length of stay (days)	98	10·8 ±7·2	10·0 ±7·2	0·59	98	7·9 ±4·2	11·0 ±7·2	0·17
Noninvasive ventilation	108	8 (12·1)	7 (16·7)	0·07	98	1 (6·7)	14 (16·9)	0·45
Transfer into intensive care unit	108	9 (13·6)	5 (11·9)	0·79	98	1 (6·7)	13 (15·7)	0·69
Mortality	108	1 (1·5)	4 (9·5)	0·07	98	0 (0·0)	5 (6·0)	1·00

Continuous variables are expressed as mean ± standard deviation. Categorical variables are expressed as number of patient (percentage). N: number of available data

* Malnutrition: body mass index (BMI) < 18·5 kg/m² (or < 21 kg/m² if age was ≥ 70 years) or weight loss ≥ 5% in one month, and/or weight loss ≥ 10% in six months

** Nutritional risk is defined as a Nutritional Risk Index (NRI) < 97·5. NRI = 1·519 x albumin (g/l) + 0·417 x (measured weight/ usual weight) x 100.
